# *Clostridioides difficile* Biology: Sporulation, Germination, and Corresponding Therapies for *C. difficile* Infection

**DOI:** 10.3389/fcimb.2018.00029

**Published:** 2018-02-08

**Authors:** Duolong Zhu, Joseph A. Sorg, Xingmin Sun

**Affiliations:** ^1^Department of Molecular Medicine, Morsani College of Medicine, University of South Florida, Tampa, FL, United States; ^2^Department of Biology, Texas A&M University, College Station, TX, United States

**Keywords:** *C. difficile*, spores, germination, CDI, sporulation

## Abstract

*Clostridioides difficile* is a Gram-positive, spore-forming, toxin-producing anaerobe, and an important nosocomial pathogen. Due to the strictly anaerobic nature of the vegetative form, spores are the main morphotype of infection and transmission of the disease. Spore formation and their subsequent germination play critical roles in *C. difficile* infection (CDI) progress. Under suitable conditions, *C. difficile* spores will germinate and outgrow to produce the pathogenic vegetative form. During CDI, *C. difficile* produces toxins (TcdA and TcdB) that are required to initiate the disease. Meanwhile, it also produces spores that are responsible for the persistence and recurrence of *C. difficile* in patients. Recent studies have shed light on the regulatory mechanisms of *C. difficile* sporulation and germination. This review is to summarize recent advances on the regulation of sporulation/germination in *C. difficile* and the corresponding therapeutic strategies that are aimed at these important processes.

## Introduction

*Clostridioides difficile* (formerly *Clostridium difficile*; Lawson et al., 2016; Oren and Garrity, 2016) is a Gram-positive, spore-forming, toxin-producing, anaerobic bacterium which has established itself as a leading cause of nosocomial antibiotic-associated diarrhea in the developed countries (Sebaihia et al., 2006). It is found widely in the mammalian gastrointestinal (GI) tract and can cause toxin-mediated *C. difficile* infections (CDI) that range from mild diarrhea to pseudomembranous colitis and potential death (Lessa et al., 2012). *C. difficile* causes over 500,000 infections per year in the United States alone, resulting in an estimated 29,000 deaths and an estimated cost of $1–3 billion (Dubberke and Olsen, 2012; Lessa et al., 2015). Currently, antibiotics are the standard treatments for CDI (i.e., vancomycin, metronidazole, or fidaxomicin; Evans and Safdar, 2015). Though effective, CDI recurrence after the initial treatment can still reach up to 15–35% in treated patients (Leffler and Lamont, 2015). Though recurrence is not fully understood, one of the reasons for high recurrence rate is that *C. difficile* spores may still be present within the patients gut and germinate to the vegetative form after completion or discontinuation of antibiotic treatment (Cornely et al., 2012). Meanwhile, poor host immune response to *C. difficile* and frequent disruption of the normal gut flora may also contribute to the high recurrence rate (Johnson, 2009). Due to the inherent antibiotic resistance of *C. difficile* cells and high prevalence of CDI in some hospitals, the Centers for Disease Control and Prevention (CDC) has listed *C. difficile* as “an urgent threat” regarding the antibiotic associated threats to the United States (Centres for Disease Control and Prevention (US), 2013).

Because *C. difficile* is an obligate anaerobic pathogen, the vegetative cells are unable to survive outside of a host in the aerobic environment. When *C. difficile* cells meet certain environmental stimuli (e.g., nutrient deprivation, quorum sensing, and other unidentified stress factors), they will initiate a sporulation pathway to produce sufficient dormant spores to survive in extreme situations (Setlow, 2006; Rodriguez-Palacios and LeJeune, 2011; Deakin et al., 2012; Higgins and Dworkin, 2012). *C. difficile* pathogenesis relies on the formation of aerotolerant dormant spores which allows *C. difficile* to persist within the host and to disseminate through patient-to-patient contact/environmental contamination (Britton and Young, 2012). In the host GI tract, the dormant spores must germinate from dormancy to form the actively growing vegetative cells which produce the toxins that cause the primary symptoms of the disease. Under suitable conditions, when germinant receptors sense the presence of small molecules (germinants), spore germination will be induced (Sorg and Sonenshein, 2008).

Recent studies have focused on the regulatory mechanisms of *C. difficile* sporulation/germination to gain insight into these important processes. However, when compared to other well-studied organisms such as *Bacillus subtilis* and *Clostridium perfringens*, our knowledge of *C. difficile* spore biology still lags far behind. In this review, we will discuss recent progresses in the field of *C. difficile* spore biology, specifically on the sporulation and germination processes and their implications for CDI treatment.

## *C. difficile* sporulation

### Sporulation program

**Figure 1:**
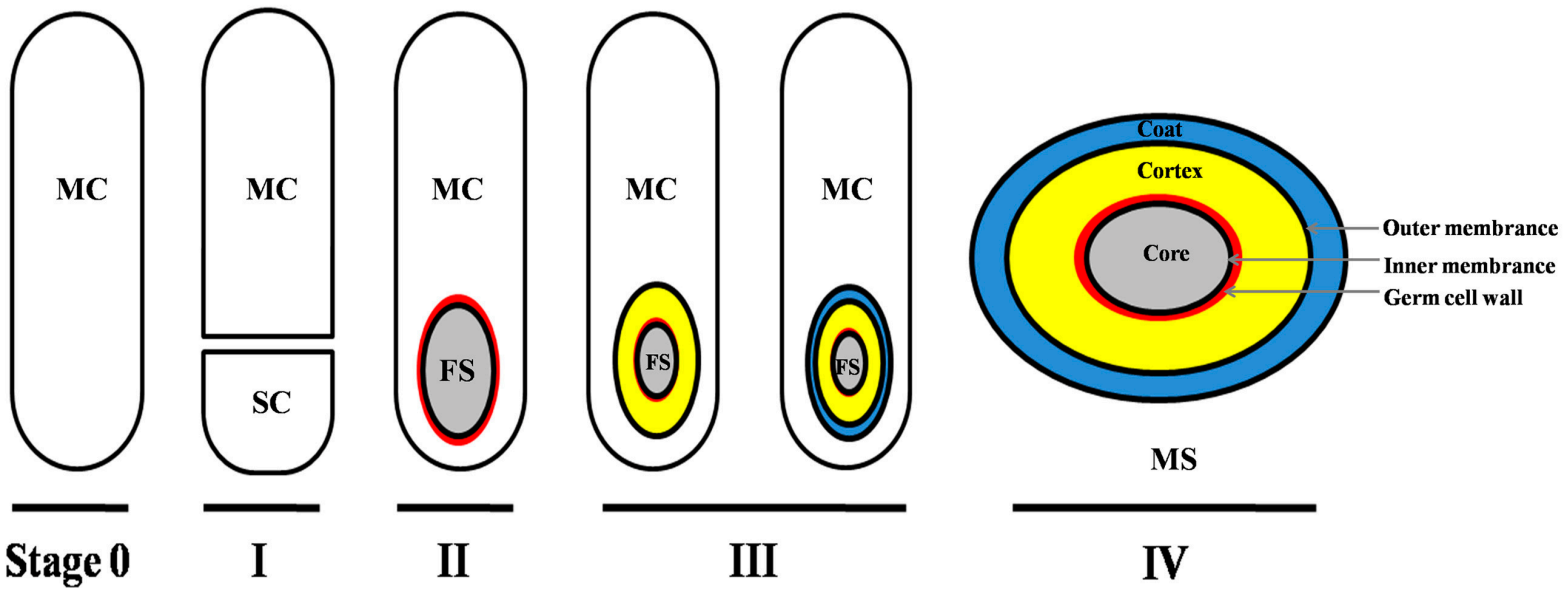
Main morphogenetic stages of the sporulation process and structure of *C. difficile* spore. This figure was drawn based on the references (Edwards and McBride, 2014; Gil et al., 2017). The main layers of spore structure are shown in this figure (IV). Some *C. difficile* strains have exosporium, while some have not. The exosporium structure was not shown in this figure. MC, mother cell compartment; SC, smaller compartment; FS, forespore compartment; MS, mature spore.

Though the signals/molecules that trigger *C. difficile* sporulation have not been identified, based on studies in other organisms, it is likely that environmental stimuli such as nutrient limitation, quorum sensing, and other unidentified stress factors are involved (Higgins and Dworkin, 2012). In fact, though the mechanism is not well-defined, a recent report has suggested that quorum sensing is important for *C. difficile* spore formation (Darkoh et al., 2016). As described in other spore-forming bacteria (e.g., *B. subtilis*), the main process of *C. difficile* sporulation contains four morphogenetic stages (Figure 1; Edwards and McBride, 2014; Gil et al., 2017): (I) an asymmetric septation generates a smaller compartment (SC) and a larger mother cell (MC); (II) the MC engulfs the SC (now the forespore) in a phagocytic-like event resulting in a forespore being wholly contained within the MC's cytoplasm; (III) the spore cortex and coat layers are assembled; (IV) the MC lyses and releases the mature spore into the surrounding environment. Though the mechanisms that initiate spore formation may differ between organisms, the overall spore architecture is conserved among endospore-forming bacteria. Located in the center of the mature spore is the core. The spore core contains the genomic DNA, mRNA, ribosomes, protein, and is very rich in pyridine-2,6-dicarboxylic acid (DPA), commonly as a calcium salt (CaDPA). The spore core is surrounded by an inner membrane, a peptidoglycan-containing germ cell wall, a specialized peptidoglycan-containing cortex, an outer membrane and layers of coat protein (Figure 1; Edwards and McBride, 2014; Gil et al., 2017). In some *C. difficile* strains, an exosporium layer surrounds the coat, but not all spore-forming bacteria and not all *C. difficile* strains have this layer (thus this layer is not shown in Figure 1).

### Regulator CodY and CcpA

**Figure 2:**
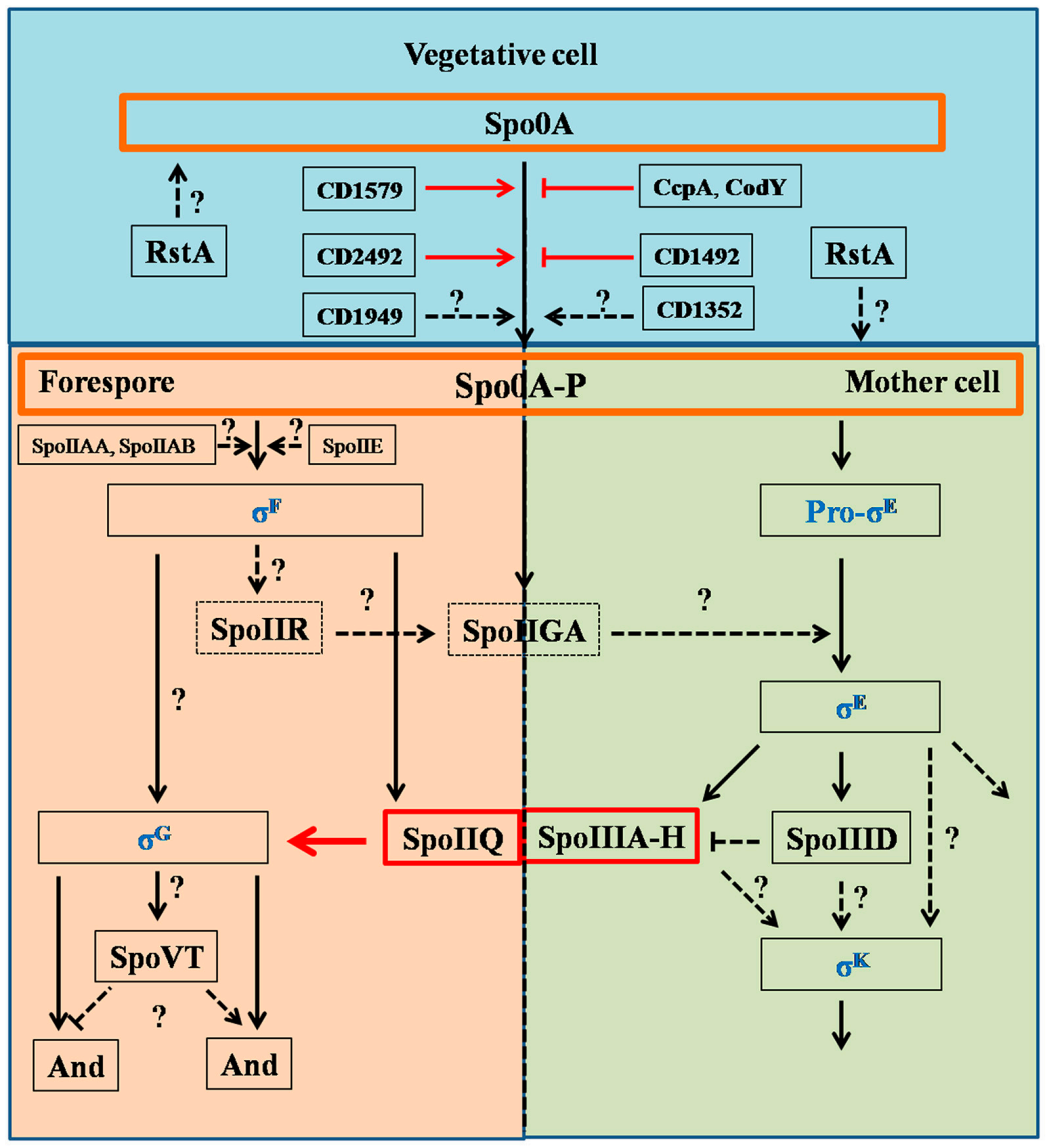
Regulation pathways of *C. difficile* sporulation. This figure was drawn based on the references (Fimlaid et al., 2013; Fimlaid and Shen, 2015). Regulation of box SpoIIQ, SpoIIIA-H, CD1579 (CD630_15790), CD1492 (CD630_14920), CD2492 (CD630_24920), CcpA, CodY, and RstA were added in this figure based on the recent advances in *C. difficile* sporulation. CD1492 (CD630_14920), CcpA, and CodY were the negative regulators in sporulation pathways. Function of SpoIIQ-SpoIIIAH complex was characterized by Pinho group, recently (Serrano et al., 2016). Spo0A and Spo0A-P were scheduled in orange boxes, four sigma factors were colored in blue. Dashed boxes indicate that the function of the proteins in regulation pathways has not been identified. Black arrows indicate the regulatory relationship between the factors has been confirmed, dashed arrows indicate the regulatory relationship between the factors has not been tested, red arrows, stops and boxes indicate the function of proteins and the correlation of factors has been confirmed recently. Question marks indicate that there is suggestive, but no conclusive experimental evidence.

Environmental stimuli (e.g., nutrient deprivation or quorum sensing) could trigger *C. difficile* sporulation. Previous studies in *Bacillus* and *Clostridioides* species have revealed that the CodY and CcpA nutritional sensor proteins work as negative regulators of sporulation (Figure 2; Duncan et al., 1995; Hofmeister et al., 1995; Karow et al., 1995; Londoño-Vallejo and Stragier, 1995; Antunes et al., 2012; Nawrocki et al., 2016; Serrano et al., 2016). Among the genes CodY regulates are genes involved in spore formation including *spo0A, rapA, rapC, rapE, sinI/R, sigH*, and *kinB*. Recently, Edwards et al. demonstrated that the oligopeptide permease genes *app* and *opp*, and the putative sporulation regulator genes *sinI* and *sinR*, were regulated by CodY to suppress the initiation of *C. difficile* sporulation (Edwards et al., 2014). Previous studies indicated that the variability of CodY-dependent regulation is an important contributor to virulence and sporulation in current epidemic isolates (Bennett et al., 2007; Majerczyk et al., 2008; van Schaik et al., 2009). But, to date, the regulatory mechanisms by which CodY affects sporulation are not fully understood because the factors that initiate sporulation in *C. difficile* are still being identified.

CcpA, a LacI family DNA-binding transcriptional regulator, works as a global transcriptional regulator that responds to the availability of carbohydrates (Deutscher et al., 2006). The CcpA sequence and structure are conserved in *C. difficile*, and has high homology to other pathogens (identity ≥62% analyzed with NCBI website), such as *Staphylococcus aureus, Clostridium perfringens*, and *Clostridium perfringens*. CcpA represses the use of alternative carbon sources and positively regulates sugar uptake, fermentation, and amino acid metabolism (Fujita, 2009). In the past few years, CcpA has been shown to regulate several virulence-associated genes. For example, it regulates the expression of the *S. aureus* α-hemolysin (*hla*), enterotoxins A, B, and C (*sea, seb*, and *sec*) genes, the *C. perfringens* enterotoxin (*cpe*) gene, and the *Bacillus anthracis atxA* and protective antigen (*pagA*) genes (Varga et al., 2004; Seidl et al., 2006; Chiang et al., 2011). Moreover, CcpA also plays critical role in the control of colonization, antibiotic resistance, and biofilm formation (Seidl et al., 2006; Varga et al., 2008). In *C. difficile*, CcpA directly regulates the PaLoc genes (*tcdR, tcdB, tcdA*, and *tcdC*) to mediate glucose-dependent repression of toxin production and indirectly regulates *C. difficile* sporulation (Antunes et al., 2011).

### Sporulation progress

Studies have revealed the master transcriptional regulator Spo0A plays the critical role during *C. difficile* sporulation (Deakin et al., 2012). In all studied endospore-forming bacteria, Spo0A must be phosphorylated (Spo0A-P) by a histidine kinase to become activated. In *Bacilli*, these histidine kinases (Kin) are found on the plasma membrane and lead to the phosphorylation of Spo0A through Spo0F/Spo0B phosphotransfer system. *C. difficile* does not encode orthologs of these kinases or the phosphotransfer system. However, previous studies have demonstrated five putative orphan histidine kinases {CD1352 [CD630_13520; *cprK* (McBride and Sonenshein, 2011)], CD1492 (CD630_14920), CD1579 (CD630_15790), CD1949 (CD630_19490), and CD2492 (CD630_24920)} in *C. difficile* strain 630 genome that could potentially phosphorylate Spo0A (Figure 2; Underwood et al., 2009). A ClosTron mutation in CD2492 (CD630_24920) resulted in a decreased capacity of the resulting strain to generate spores compared to the WT parent. However, this mutant still generated spores (~4%) suggesting that other histidine kinases can phosphorylate Spo0A or lead to Spo0A phosphorylation (Underwood et al., 2009). In support of this hypothesis, CD1579 (CD630_15790) was shown to autophosphorylate and transfer a phosphate directly to Spo0A (Underwood et al., 2009). Importantly though, the authors did not complement their CD2492 ClosTron mutation, which could have polar effects on downstream genes. In contrast, Childress et al. found that a markerless deletion of CD1492 (CD630_14920) was an inhibitor of sporulation and suppresses spore formation (Childress et al., 2016). This phenotype could be complemented by expression of the wild type allele. Currently, the function of the other putative orphan histidine kinases and their ability to phosphorylate Spo0A are unclear.

Recently, RstA was found to be a novel, positive regulator of sporulation initiation in *C. difficile* (Figure 2; Edwards et al., 2016). RstA positively affects the initiation of *C. difficile* sporulation through its peptide-interacting domain (TPR), and negatively regulates toxin production and mobility by affecting the flagellar-specific sigma factor (SigD) expression. But a detailed pathway on the regulation of sporulation initiation by RstA is not fully appreciated. The authors hypothesized that RstA may be a *C. difficile* global transcriptional regulator, similar to the broad physiological roles that the RNPP (Rap/NprR/PlcR/PrgX) proteins play in other bacteria (Edwards et al., 2016).

Spo0A functions as a critical regulator for sporulation by regulating sporulation-specific RNA polymerase sigma factors, especially for σ^E^, σ^F^, σ^G^, and σ^K^ (Fimlaid and Shen, 2015). These σ factors activate compartment-specific transcriptional regulation during *B*. *subtilis* sporulation and are also conserved in *Clostridium* species. σ^E^ and σ^K^ are MC-specific, and σ^F^ and σ^G^ are specific to the developing forespore. The sporulation regulatory pathway of sigma factors in *C. difficile* is illustrated in Figure 2: (1) σ^F^ is activated in the forespore soon after polar septation, and it controls early stages of development in this compartment. σ^F^ becomes active when the anti-sigma factor SpoIIAB (ADP form) binds to the anti-anti-sigma factor SpoIIAA in its unphosphorylated form, while SpoIIE catalyzes dephosphorylation; (2) σ^F^ activity leads to expression of SpoIIR, which interacts with the membrane-bound protease SpoIIGA (SpoIIGA is responsible for the cleavage of pro-σ^E^ through trans-septum signaling, yielding active σ^E^); (3) after σ^F^ and σ^E^ become specifically active in the forespore and MC, respectively, the MC engulfs the forespore; (4) σ^E^ activity leads to expression of SpoIIIA-H, which works with σ^F^-controlled SpoIIQ to form a channel in the inner and outer forespore membranes. SpoIIIAH and SpoIIQ localize to the asymmetric septum and the engulfing membranes and interact in the intermembrane space via their extracytoplasmic domains; (5) σ^E^-controlled SpoIIID activates σ^K^ in the MC (Haraldsen and Sonenshein, 2003; Fimlaid et al., 2013; Pereira et al., 2013; Paredes-Sabja et al., 2014; Saujet et al., 2014). Though many of the factors that control spore formation are conserved in *C. difficile*, there are some differences in the sporulation program between *C. difficile* and *B. subtilis*. For instance (Figure 2), pro-σ^k^ is not encoded by *C. difficile*, but the mature σ^k^ is produced directly in *C. difficile*, σ^E^ activation is dispensable for σ^G^ activation, σ^G^ activation is dispensable for σ^K^ activation, and σ^K^ is responsible for transcribing the germinant receptors while σ^G^ is responsible in *B*. *subtilis* (Fimlaid et al., 2013; Pereira et al., 2013). Importantly, the FS line of gene expression occurs largely independently of the MC line of gene expression. Moreover, σ^G^ can be activated before σ^E^ and σ^K^, indicating that the order/sequence of sigma factor activation is not as tightly controlled in *C. difficile* as it is in *B. subtilis*.

Finally, and in another departure from the model of spore formation in *B. subtilis*, a recent article by Ribis and colleagues used a TargeTron-based gene disruption demonstrated that the SpoVM protein is not required for spore formation/maturation (Ribis et al., 2017). SpoVM is a small protein that is expressed in the MC that recognizes the positive curvature of outer membrane of the developing forespore and embeds itself there. In *B. subtilis*, SpoVM recruits the SpoIVA scaffolding protein which polymerizes and surrounds the forespore. Subsequently, the coat is deposited onto the polymerized SpoIVA protein. In *C. difficile*, a *spoVM* mutation resulted in a modest defect in spore production (< 5-fold), but their resistance properties are not different from a wildtype spore. This phenotype could be complemented through chromosomal complementation of the wild type allele. However, and importantly, the mutation in *spoVM* lead to a mislocalization of the coat proteins to one pole of the developing forespore and the coat extended into the MC cytoplasm; SpoIVA still polymerized on the surface of the forespore.

## *C. difficile* spore germination

### Germination program

In most organisms, spore germination is induced when specific germinant receptors sense the presence of small molecules (germinants; Setlow, 2003). To date, germination has been most-studied in *Bacillus* spp. and it contains three main steps (Paredes-Sabja et al., 2011, 2014): (I) germinant (e.g., nucleosides, sugars, amino acids, and/or ions) binding with their cognate Ger-type receptors (GerAA-AB-AC) at the inner spore membrane to trigger the release of monovalent cations (H^+^, Na^+^, and K^+^) and the large amount of CaDPA stored within the core, in exchange for water; (II) CaDPA release and core rehydration leads to the activation of spore cortex lytic enzymes (SCLEs) SleB and CwlJ; (III) activated SleB and CwlJ degrade the peptidoglycan cortex layer, which allows for full core rehydration and resumption of metabolism in the spore core.

### Germinant recognition/signaling

**Figure 3:**
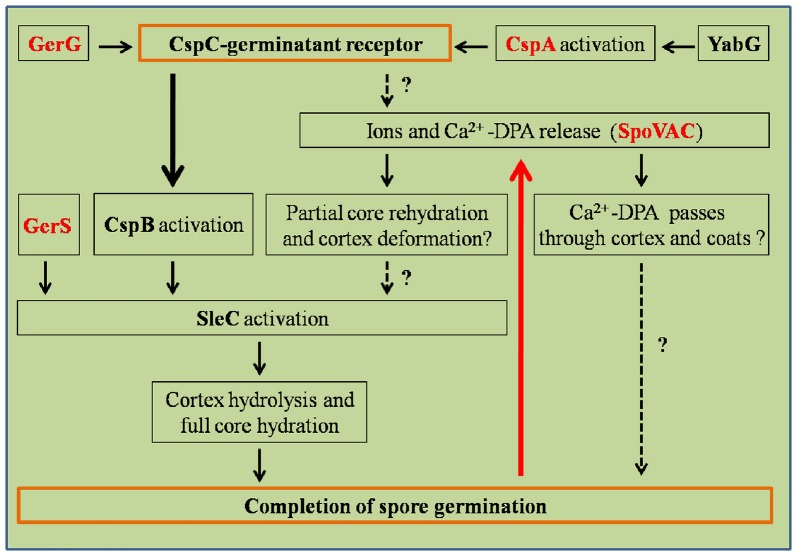
Regulation pathways of *C. difficile* spore germination. This figure was drawn based on the references (Paredes-Sabja et al., 2011; Fimlaid et al., 2013). Regulation of box GerG, GerS, CspA, and SpoVAC texted in red were drawn in this figure based on the recent advances in *C. difficile* spore germination. GerS, GerG, and SpoVAC proteins were characterized by Shen group, recently (Fimlaid et al., 2015; Donnelly et al., 2016, 2017). CspC-germinant receptor and completion of germination were scheduled in the orange boxes. Black arrows indicate the regulatory relationship between the factors has been confirmed, dashed arrows indicate the regulatory relationship between the factors has not been tested. Thick black/red arrow indicates central signal pathway in germination progress. Question marks indicate that there is suggestive, but no conclusive experimental evidence.

Germination of *C. difficile* spores is the first step for initiating CDI. *C. difficile* spore germination is activated in response to certain host-derived bile salt germinants [e.g., taurocholic acid (TCA)/cholic acid derivatives] and amino acids (e.g., glycine or alanine; Sorg and Sonenshein, 2008). Chenodeoxycholic acid-derivatives (a compound structurally similar to cholic acid but lacking the 12α-hydroxyl group) are competitive inhibitors of cholic acid-mediated germination (Francis et al., 2013b). Though the Ger-type germinant receptors have been widely studied in many organisms, including *C*. *perfringens* and *Clostridium botulinum/sporogenes, C. difficile* does not encode orthologs of the *gerA* germinant. Instead, *C. difficile* spores use the subtilisin-like, CspC pseudoprotease as the bile acid germinant receptor (Figure 3; Paredes-Sabja et al., 2008; Francis et al., 2013a, 2015; Wang S. W. et al., 2015; Bhattacharjee et al., 2016; Francis and Sorg, 2016). *C. difficile* packages three subtilisin-like serine proteases proteins, CspA, CspB, and CspC, into the spore. In *C. difficile*, CspB, and CspA are encoded as a *cspBA* gene fusion, where the CspA portion of CspBA lacks an intact catalytic triad (Adams et al., 2013; Kevorkian et al., 2016). The CspBA fusion protein undergoes interdomain cleavage during spore formation, leading to the separation of CspB and CspA, which are transported into the spore by unknown mechanisms. *cspC* is encoded downstream of *cspBA* and, similar to *cspA*, encodes an incomplete catalytic triad. Despite the loss of apparent catalytic activity, *cspC* (and *cspA*) important for *C. difficile* spore germination. Interruption of the *cspC* coding region through ethyl methanesulfonate (EMS)-generated SNPs and TargeTron methods abrogates spore germination, and certain SNPs in the *cspC* sequence also affect germinant specificity (Francis et al., 2013a). Similarly, though *cspA* lacks an intact catalytic triad, *cspA* is essential for spore germination by controlling the levels of CspC into the developing spore (Francis et al., 2013a; Kevorkian et al., 2016). Only CspB contains an intact catalytic triad and, thus, is hypothesized to be important for activating the SCLE, pro-SleC, to its active, cortex-degrading form (Kevorkian et al., 2016).

Activation of the cortex hydrolase SleC depends on the CspB protease, which cleaves the N-terminal pro sequence from the protein. Activated SleC degrades the cortex leading to CaDPA release from the spore core in response to osmotic swelling sensed at the inner spore membrane as a result of cortex degradation (Francis and Sorg, 2016). The osmotic pressure at the inner spore membrane is regulated by SpoVAC (a mechanosensing protein), which allows CaDPA release from the core (Velásquez et al., 2014; Donnelly et al., 2016; Francis and Sorg, 2016). Strikingly, inactivation of either the CspC or SleC inhibited cortex degradation and CaDPA release. These results suggest that the CspC is required for CaDPA release and that cortex degradation precedes CaDPA release, opposite to what occurs in *B. subtilis* (Francis and Sorg, 2016). These studies suggest that the process of *C. difficile* spore germination appears to occur in an outside-in manner, while in *B*. *subtilis*, the signal appears to travel from the inside-out (Francis and Sorg, 2016).

### GerG and GerS regulators of spore germination

Recently, GerG and GerS were identified as important players in *C. difficile* spore germination (Figure 3; Fimlaid et al., 2015; Donnelly et al., 2017). Donnelly et al. identified the *C. difficile*-specific protein GerG as an important player in the *C. difficile* germination process (Donnelly et al., 2017). A deletion of *C. difficile gerG* resulted in spores with germination defects and reduced responsiveness to bile salt germinants. This phenotype was likely due to the decrease in the incorporation of the CspC, CspB, and CspA germination proteins into spores; this phenotype could be complemented *in trans*. Similarly, Fimlaid et al. identified another regulator of *C. difficile* spore germination using TargeTron-based gene disruption (Fimlaid et al., 2015). The GerS lipoprotein functions as a critical regulator in *C. difficile* spore germination (Fimlaid et al., 2015). In this study, the *gerS* mutant has a severe germination defect and fails to degrade cortex; this phenotype could be complemented *in trans*. Interestingly, *C. difficile gerS* mutant spores still cleave pro-SleC to its active form, suggesting that either cortex is not appropriately modified for SleC-recognition or that SleC is bound to other proteins that GerS regulates (Fimlaid et al., 2015). Importantly, loss of GerS attenuated the *C. difficile* virulence in the hamster infection model (Fimlaid et al., 2015). Because GerG and GerS are found exclusively in *C. difficile*, GerG and GerS proteins could be the potential targets to develop *C. difficile*-specific anti-infective therapies.

### Activators and inhibitors of *C. difficile* spore germination

Bile-acid mediated germination is essential for *C. difficile* spore germination and CDI in mammalian GI tract. Bile acids are the end products of cholesterol metabolism in liver and are essential for lipoprotein, glucose, drug, and energy metabolism (Chiang, 2009; Howerton et al., 2011). In humans, cholic acid (CA) and chenodeoxycholic acid (CDCA) are two main primary bile acids (PBAs) that are conjugated with either taurine or glycine. Though most of the bile acids secreted into the gut are reabsorbed and recycled back to the liver to be used in other rounds of digestion, some escape hepatic recirculation and enter the large intestine where they become acted upon by the colonic microbiome. Here, the conjugated bile acids become deconjugated due to the action of bile salt hydrolases that are expressed on the cell surfaces of many different bacteria. Subsequently, a small subset of the colonic microbiome will take up and 7α-dehydroxylate the PBAs to form secondary bile acids (SBAs; Ridlon et al., 2006). About 50 different chemically distinct SBAs [e.g., deoxycholate (DCA), lithocholate (LCA), ursodeoxycholate (UDCA), isodeoxycholate (iDCA), and isolithocholate (iLCA)] can be found in human large intestine (Setchell et al., 1983). Recently, Thanissery et al. have analyzed the impact of gut microbial derived SBAs on *C. difficile* life cycle, specifically, the differences in inhibition efficiency of spore germination, growth, and toxin activity among of DCA, iDCA, LCA, iLCA, UDCA, ωMCA, and HDCA in clinically relevant *C. difficile* strains R20291 and CD196 (ribotype 027), M68 and CF5 (017), 630 (012), BI9 (001), and M120 (078) (Thanissery et al., 2017). Not surprisingly, the authors found these cholic acid- and chenodeoxycholic acid-derivatives all impacted the *C. difficile* life cycle; the sensitivity varied by strain and SBA.

Although bile acids are essential to activate *C. difficile* spore germination, they are not sufficient to activate germination on their own. Amino acid co-germinants are also required for spore germination (Sorg and Sonenshein, 2008; Howerton et al., 2011; Shrestha and Sorg, 2017; Shrestha et al., 2017). However, different amino acids function as co-germinants with different spore germination efficiencies. Glycine is the most effective co-germinant in *C. difficile*, while alanine is most-often used as co-germinant in *B. subtilis* and other organisms. In *B. subtilis*, L-alanine interacts with the GerAA-AB-AC germinant receptor to trigger CaDPA release from the spore core and subsequent cortex hydrolysis. However, D-alanine competitively-inhibits L-alanine-mediated spore germination in *B. subtilis* (Yasuda and Tochikubo, 1984). In *C. difficile*, L-alanine can also function as a co-germinant with TCA to stimulate spore germination (Shrestha et al., 2017). Though D-alanine is unable to inhibit L-alanine-mediated *C. difficile* spore germination, unlike what is observed in *B*. *subtilis*, D-alanine can work as a co-germinant to trigger *C. difficile* spore germination in defined medium (Shrestha and Sorg, 2017; Shrestha et al., 2017). In order for D-alanine to function as a good co-germinant, an alanine racemase (Alr2) should be present in the *C. difficile* spore. Alr2 interconverts L-alanine and D-alanine (Shrestha et al., 2017). Interestingly, *C. difficile* Alr2 can also interconvert L- and D-serine, and both of these amino acids can act as co-germinants for *C. difficile* spore germination (Shrestha et al., 2017). Building on this work, Shrestha et al. found that many different amino acids are co-germinants when tested at 37°C (Shrestha and Sorg, 2017). In this work, two different *C. difficile* strains responded to a hierarchy of amino acid co-germinants. For UK1 and M68 strains, glycine was the most effective co-germinant (EC_50_ = ~200 μM) and L-alanine, taurine, and L-glutamine were also good co-germinants (Shrestha and Sorg, 2017). Interestingly, amino acids that regulate important physiological processes were not co-germinants (L-isoleucine, L-leucine and L-valine).

Recently, Kochan et al. identified a critical role for Ca^2+^ during *C. difficile* spore germination (Kochan et al., 2017). In their study, they found that *C. difficile* spores cannot germinate in rich medium supplemented with TCA but without Ca^2+^, indicating that Ca^2+^ is indispensable for spore germination. The authors suggested that it works together with glycine to stimulate germination; however, Ca^2+^ may play a role in the activity of the CspB serine protease, the CspC germinant receptor, the CspA pseudoprotease, or in the activity of the cortex hydrolase. Other subtilisin-like proteases require Ca^2+^ for activity (Siezen and Leunissen, 1997) and some cortex-degrading enzymes also require Ca^2+^. Though no Ca^2+^ was found in the CspB crystal structure, the structures of CspC and CspA have yet to be determined. Thus, Ca^2+^ may not function as a co-germinant with glycine, but, rather, as an essential cofactor for *C. difficile* spore germination. However, and importantly, the role of Ca^2+^ during *C. difficile* spore germination was also verified in the murine model. *Ex vivo* assays with mouse ileal contents that were depleted with chelex resin (to remove Ca^2+^) did not support germination of *C. difficile* spores (Kochan et al., 2017). This work provided a novel potential strategy for CDI control by modulating intestinal Ca^2+^ concentration.

In summary, although several main components of spore sporulation/germination machinery of *C. difficile* have been identified and characterized, several questions remain regarding how *C. difficile* decides when to enter the sporulation pathway. Moreover, though the Csp pseudoproteases are important for germination, how they interact with and transmit the bile acid signal are still unknown. Further detailed work is necessary to characterize these important aspects of *C. difficile* physiology.

## Treatments of CDI based on sporulation/germination

Currently, the standard treatment of CDI is the use of vancomycin, metronidazole, or fidaxomicin, each of which has some level of recurring disease due to the continued insult to the colonic microbiome and the presence of spores within the colon/environment (Allen et al., 2013). To meet this challenge, non-antibiotic and immune-based therapies against CDI have been developed, such as anti-toxins, vaccines, fecal microbiota transplant (FMT), and anti-germination-based compounds (Gerding et al., 2008; Howerton et al., 2013; Kociolek and Gerding, 2016). Many anti-toxins and vaccines for CDI have been developed in the past two decades (Cox et al., 2013; Monteiro et al., 2013; Mathur et al., 2014; Zhao et al., 2014; Wang Y. K. et al., 2015; Yang et al., 2015; Qiu et al., 2016). Though these treatments can effectively decrease the morbidity and mortality of CDI, most of the anti-toxins and vaccines cannot suppress *C. difficile* colonization and kill *C. difficile* spores. Therefore, with these treatments, there are still risks of potential CDI relapse in the host.

Instead of merely neutralizing *C. difficile* toxins in host, strategies which can directly decrease *C. difficile* colonization, kill the vegetative cells, and suppress sporulation/germination are desirable treatments for CDI. FMT is an effective strategy to reconstruct the gut microbiota to suppress *C. difficile* colonization, especially for patients who have multiple bouts of recurring disease and who have failed conventional treatment methods (Borody and Khoruts, 2012; Weingarden et al., 2014; Khoruts and Sadowsky, 2016; Kim et al., 2016). Although FMT is deemed relatively safe and low-cost, the unappealing aesthetics of the procedure is often a concern of patients (Sampath et al., 2013; Varier et al., 2015). Because the *C. difficile* spore form is necessary for dissemination and persistence, sporulation/germination are critical steps for CDI. Thus, it is worth developing therapeutic strategies for disrupting *C. difficile* disease transmission and spread according to *C. difficile* spore biology. Basing on the progress of *C. difficile* spore germination, the PBA CDCA and secondary bile acids LCA, UDCA, and iLCA are potent inhibitors of *C. difficile* spore germination (Sorg and Sonenshein, 2010; Zhang and Klaassen, 2010; Heeg et al., 2012). Moreover, several mouse-derived bile acids, such as α-muricholic acid, β-muricholic acid, and ω-muricholic acid inhibit *C. difficile* spore germination and growth (Francis et al., 2013b). Excitingly, synthesized bile acid analogs, such as CAmSA, methylchenodeoxycholic acid diacetate, and compound 21b (derived from UDCA) have been identified to inhibit *C. difficile* spore germination (Sorg and Sonenshein, 2009; Howerton et al., 2013; Stoltz et al., 2017). Of these compounds, CAmSA showed promise in inhibiting/delaying *C. difficile* disease in a mouse model of CDI. These anti-germination-based strategies could work in a couple of different ways. (i) High risk patients who are to be treated with antibiotics could also take an anti-germinant to prevent the germination of spores within the host's gut. This patient continues to take the anti-germinant during and post-antibiotic treatment so that the normal, colonic, microbiome has a chance to repopulate and provide natural protection against CDI. (ii) Patients with CDI could take the recommended course of antibiotics plus the anti-germinants. This strategy would prevent recurring disease by allowing the microbiome to re-establish colonization resistance post-antibiotic treatment. Because both strategies block germination, and thus downstream events (vegetative growth, toxin production, and spore formation), anti-germination therapy would limit the presence of spores within the surrounding environment because *C. difficile* would not have a chance to expand in population and produce spores. In contrast germination-inducing strategies are a viable option for environmental cleanup; inducing *in vivo* germination has the potential for toxin-production and, thus, exacerbation of symptoms. Due to the inherent nature of the dormant spore, harsh chemicals (e.g., bleach) are required to clean environmental surfaces. But by germinating the spores in the environment, the germinated spores become susceptible to a wider range of sanitizing agents (Nerandzic and Donskey, 2010, 2013, 2017; Nerandzic et al., 2016). More studies should be investigated for further application of germination inhibitors.

## Concluding and remarks

Although much has been learned about the sporulation/germination processes of *C. difficile* and the different therapeutic strategies for CDI, many key questions related to regulation pathways of sporulation/germination processes remain unanswered. Thus, much work remains to be done to further understand *C. difficile* spore biology and develop new efficient approaches for CDI treatment: (1) It is expected that further work will allow us to fully understand the mechanisms of the initiation of sporulation by identifying the proteins that are involved in Spo0A phosphorylation; (2) Due to the relevance of spore germination with CDI progression, it is worth defining how the bile acid germinant receptor, CspC, and the unidentified glycine germinant receptor regulate CaDPA release and cortex degradation; (3) More alternative therapeutic strategies for CDI disease need to be developed based on the knowledge of *C. difficile* sporulation/germination.

## Author contributions

All authors listed, have made a substantial, direct, and intellectual contribution to the work; DZ wrote and revised this manuscript; JS and XS revised this manuscript.

### Conflict of interest statement

The authors declare that the research was conducted in the absence of any commercial or financial relationships that could be construed as a potential conflict of interest.
